# Analysis of rod-cone dystrophy genes reveals unique mutational patterns

**DOI:** 10.1136/bmjos-2022-100291

**Published:** 2022-12-13

**Authors:** Lama Jaffal, Mariam Ibrahim, Said El Shamieh

**Affiliations:** 1 Department of Biological and Chemical Sciences, School of Arts and Sciences, Lebanese International University, Nabatyeh, Lebanon; 2 Rammal Hassan Rammal Research Laboratory, PhyToxE Research Group, Faculty of Sciences, Lebanese University, Nabatyeh, Lebanon; 3 Department of Medical Laboratory Technology, Faculty of Health Sciences, Beirut Arab University, Beirut, Lebanon

**Keywords:** genotype, INDEL mutation, biomarkers

## Abstract

**Background:**

Rod-cone dystrophy (RCD) is the most common inherited retinal disease that is characterised by the progressive degeneration of retinal photoreceptors. RCD genes classification is based exclusively on gene mutations’ prevalence and does not consider the implication of the same gene in different phenotypes. Therefore, we first investigated the mutations occurrence in autosomal recessive RCD (arRCD) and non-arRCD conditions. Then, finally, we identified arRCD enriched mutational patterns in specific genes and coding exons.

**Methods and results:**

The mutations patterns differed according to arRCD (p=0.001). Specifically, When compared with missense; insertions/deletions (OR=1.2, p=0.007), nonsense (OR=1.2, p=0.014) and splice-site mutations (OR=1.6, p=0.038) increased the OR of arRCD by 20%–60% versus non-arRCD conditions. The gene-based analysis identified that *EYS, IMPG2, RP1L1* and *USH2A* mutations were enriched in arRCD (p<0.05). The exon-based analysis revealed specific mutation patterns in exons of *CRB1*, *RP1L1* and exons 12, 60 and 62 coding for Laminin EGF and FTIII domains of *USH2A*.

**Conclusion:**

The current analysis showed that many aRCD genes have unique mutational patterns.

**STRENGTHS AND LIMITATIONS OF THIS STUDY**

- The current study is the first to investigate all the autosomal recessive rod-cone dystrophy (arRCD) genes to report unique arRCD mutational signatures in exons and genes.
- Our study has several limitations: (1) Our analysis relied on the number of reported mutations and not the patients carrying them; thus, we could not use the allelic frequencies in all our analysis; (2) No association with specific clinical ocular phenotypes such as the visual field, the electroretinogram and the fundus appearance was performed. Unfortunately, this was not possible in the current study because of the absence of this information; (3) We could not stratify these genotype–phenotype correlations according to the geographic location and (4) For the gene based, 1 test per gene was performed (63 independent tests in total); thus, a Bonferroni correction might further be used. If applied, USH2A and CRB1 remain highly associated (p<0.001). In the exon-based analysis, one test was performed for all the gene exons, thus abolishing the concern of multiple testing.

## Introduction

Rod-cone dystrophy (RCD), also known as retinitis pigmentosa, is an inherited retinal disease (IRD) characterised by the progressive degeneration of the rod and cone photoreceptors.^1^ In the most cases, this deterioration results in night blindness followed by progressive centripetal constriction of the visual field.^2^

The worldwide prevalence of RCD is around 1:4000 individuals.^2^ This condition is transmitted as a Mendelian trait caused by disease-causing mutation(s) in gene(s) associated with the disease phenotype.^2^ RCD is exceptionally heterogeneous^3^ with mutations in more than 60 genes being implicated (list of genes is accessible on: https://web.sph.uth.edu/RetNet/sum-dis.htm%23A-genes).^3 4^ Different mutations in the same gene may cause different retinal phenotypes (such as Usher syndrome and Leber congenital amaurosis), and the same mutation may produce different retinal phenotypes even among siblings.^3^ RCD has three modes of inheritance, with the autosomal recessive (ar) being the most prevalent (50%–60%), followed by autosomal dominant (ad) (30%–40%) and X-linked patterns (5%–15%).^2 5^ Mutations in 23 genes have been related to adRCD, 36 genes to arRCD and 3 genes to X-linked RCD.^3 6^ The diagnosis of RCD is usually complex due to its noticeable heterogeneity.^3^ It depends on various investigations, including a comprehensive medical examination (visual function, multimodal retinal imaging, electrophysiology) and molecular genetic testing.^7^

Genotype–phenotype correlations in RCD and other rare diseases have largely been based on cosegregation analysis. Furthermore, RCD genes classification is based exclusively on gene mutations’ prevalence and does not consider the implication of the same gene in different IRDs. Therefore, we first investigated the occurrence of mutations according to arRCD. Then, we searched for specific mutation types highly enriched in arRCD rather than non-arRCD, such as Stargardt disease, Usher syndrome, Leber congenital amaurosis and bestrophinopathies. Finally, we identified unique mutational patterns in specific genes and coding exons.

## Methods

### Data extraction, inclusion and exclusion criteria

#### The retinal information network database

The Retinal Information Network (Retnet) is a database that provides tables of genes and loci causing IRDs.^6^ Thus, it was used to search the arRCD genes, in Retnet the disease was listed as ‘retinitis pigmentosa, ar’. In total, 63 genes were found (*ABCA4, AGBL5, AHR, ARHGEF18, ARL6, ARL2BP, BBS1, BBS2, BEST1, C2orf71, C8orf37, CERKL, CLCC1, CLRN1, CNGA1, CNGB1, CRB1, CYP4V2, DHDDS, DHX38, EMC1, EYS, FAM161A, GPR125, HGSNAT, IDH3B, IFT140, IFT172, IMPG2, KIAA1549, KIZ, LRAT, MAK, MERTK, MVK, NEK2, NEUROD1, NR2E3, NRL, PDE6A, PDE6B, PDE6G, POMGNT1, PRCD, PROM1, RBP3, REEP6, RGR, RHO, RLBP1, RP1, RP1L1, RPE65, SAG, SAMD11, SLC7A14, SPATA7, TRNT1, TTC8, TULP1, USH2A, ZNF408, ZNF513*) (https://web.sph.uth.edu/RetNet/sum-dis.htm%23A-genes, last accessed on 10 June 2021).

#### Human Gene Mutation Database database

The Human Gene Mutation Database (HGMD) is a repository for published gene mutations responsible for human inherited diseases.^8^ To retrieve the HGMD mutations, we searched for every arRCD gene by entering its symbol in the gene search tab. Genetic variations in the 63 arRCD genes were downloaded in .txt format, with information including c.DNA position, protein position, class, associated phenotype and corresponding reference (N=7382). Mutations causing ‘retinal dystrophy’ or ‘retinal degeneration’ and ‘retinal disease’ were not included in the analysis since these terms are broad and do not allow a correct diagnosis. This led to 6627 mutations (accessed: 10 September 2021). We have removed the *Rhodopsin* mutations that were reported to have a dominant effect. Furthermore, we have removed the ‘duplicate’ mutations; these are different DNA mutations that lead to the same amino acid (a.a) exchange in a gene. This filtering kept 5868 mutations. For every mutation, we added a type (missense, nonsense, insertion/deletion (InDel) or splice site) based on the HGMD annotation.

#### LOVD database

Similar to HGMD Pro, genetic variations in arRCD genes were also downloaded from the LOVD database (N=1104,^9^ accessed: 20 September 2021). To retrieve all these variations, we searched for every arRCD gene by entering its symbol in the gene search tab (https://grenada.lumc.nl/LSDB_list/lsdbs/).

#### UniProt and gene databases

All a.a domains were retrieved from the universal protein knowledgebase (UniProt) (https://www.uniprot.org/).^10^ On the other hand, the longest mRNA isoform was selected from the National Center for Biotechnology Information gene database (https://www.ncbi.nlm.nih.gov/gene). These databases provided a means to annotate the protein domain and transcript location of each genetic mutation extracted from the HGMD.

### Mutations stratified according to arRCD

Each individual mutation extracted from the HGMD database was categorised as either an arRCD or a non-arRCD (any other disease even those not related to the eye such as diabetes, hearing impairment and many others) mutation. In this analysi,s individual mutations but not their frequencies were used to create an integer count or mutation occurrence statistic. As such, if a mutation was associated with disease in more than one person in the database, it was still only counted once. However, if a mutation is genetically heterogeneous—influences more than one trait—then it was counted once for each phenotype studied here. This statistic was defined for three genomic features: (1) ‘global’ or genome wide, (2) ‘genic’ or for each gene of interest and (3) ‘exonic’ or for each exon defined for the longest transcript of each gene of interest. As a sensitivity analysis, the mutations identified in the LOVD databases were also used to derive this mutation occurrence statistic but only in a (1) ‘global’ framework. To test for differences among the arRCD and non-arRCD mutation occurrence statistics, we performed a χ^2^ test of independence. The null hypothesis was defined as an equal number of variations across all the tested categories.

### Statistical analyses

The analyses were conducted using SPSS software V.20 (SPSS). All studied variables were expressed as frequencies. The plots were generated using Origin software (OriginPro, V.8, OriginLab Corporation, Northampton, Massachusetts, USA). χ^2^ and logistic regressions, the null hypothesis of no association was rejected based on p<0.05.

## Results

**Figure 1:**
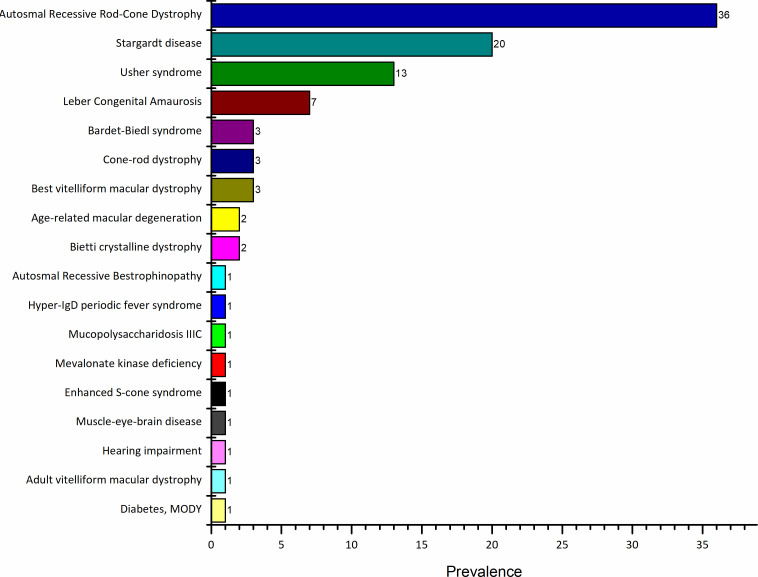
The phenotypic associations of autosomal recessive rod-cone dystrophy genotypes. The mutations data were extracted from the HGMD pro database (N=6678). A total of data were presented as percentages (%). HGMD, Human Gene Mutation Database; MODY, maturity-onset diabetes of the young.

We first used the RetNet database to identify genes previously known to cause arRCD. Sixty-three genes were found and further investigated, all listed in table 1. To study the mutation occurrence and patterns inside these arRCD genes, we searched the HGMD database, which revealed 5868 genetic variations, of which 2092 (36%) were arRCD. In comparison, the remaining two-thirds were specific for different IRDs such as Stargardt disease (17%), Usher syndrome (13%), Leber congenital amaurosis (7%), Bardet-Biedl syndrome (3%) and cone-rod dystrophy (3%) (figure 1). Interestingly, some genotypes within the arRCD genes were found in non-IRD conditions such as mucopolysaccharidosis IIIC (1%), hyper-IgD periodic fever syndrome (1%), diabetes (1%) (figure 1).

**Table 1:** Mutations occurrence and probability of causing autosomal recessive rod-cone dystrophy

Gene	arRCD	Non-arRCD	Total gene exons size (bp)	Gene size (bp, GRCh38/hg38)
	N (%)	N		
*USH2A*	415 (34)	811	18 883	800 558
*EYS*	325 (96)	13	10 589	1 987 247
*RP1*	195 (90)	22	7100	362 299
*CRB1*	149 (43)	195	5006	276 952
*PDE6B*	140 (89)	16	3403	45 210
*CNGB1*	67 (93)	5	5645	88 789
*MERTK*	66 (74)	23	3626	130 955
*ABCA4*	62 (5)	1109	7326	128 315
*PCARE*	58 (81)	12	7046	13 548
*PDE6A*	59 (94)	4	5642	86 841
*RPE65*	50 (22)	174	2608	21 133
*CNGA1*	39 (91)	4	2865	80 705
*IFFT140*	35 (40)	52	5268	101 646
*TULP1*	35 (49)	35	2094	15 023
*IMPG2*	31 (72)	12	8352	98 030
*CERKL*	29 (73)	11	3290	145 625
*MAK*	28 (100)	0	3883	75 831
*RP1L1*	24 (41)	34	7978	105 839
*PROM1*	22 (42)	31	3977	121 303
*FAM161A*	17 (85)	3	3860	53 904
*NR2E3*	18 (24)	57	2004	25 622
*BBS2*	14 (19)	61	2814	117 028
*NRL*	13 (50)	13	1974	36 349
*RLBP1*	13 (42)	18	1752	11 746
*AGBL5*	11 (92)	1	3237	28 259
*IFT172*	12 (32)	25	5329	45 429
*SPATA7*	11 (24)	31	2013	85 427
*C8ORF37*	10 (67)	5	3373	24 289
*RBP3*	10 (69)	5	4276	9519
*ARL2BP*	9 (100)	0	2100	8377
*SAG*	9 (45)	8	1749	39 240
*ZNF408*	9 (33)	18	2497	4883
*BEST1*	8 (2)	332	2679	15 695
*PRCD*	8 (100)	0	1994	25 995
*REEP6*	8 (89)	1	1373	6762
*RGR*	7 (58)	5	1475	29 295
*KIAA1549*	7 (70)	3	12 427	150 009
*LRAT*	7 (32)	14	4888	126 176
*BBS1*	5 (6)	84	3370	23 008
*CYP4V2*	5 (6)	78	4704	21 897
*POMGNT1*	5 (7)	65	2936	31 623
*ADGRA3*	4 (67)	2	4567	170 996
*ARL6*	4 (24)	13	1531	36 722
*DHDDS*	4 (44)	5	3330	39 025
*KIZ*	4 (80)	1	2201	120 648
*TRNT1*	4 (12)	28	2252	23 964
*SMAD1*	4 (67)	2	3056	78 407
*DHX38*	3 (50)	3	4470	19 300
*IDH3B*	3 (60)	2	1545	5825
*TTC8*	3 (19)	12	2203	56 927
*PDE6G*	2 (100)	0	1023	12 669
*CLRN1*	2 (6)	33	2398	46 837
*RHO*	2 (100)	0	2768	6706
*EMC1*	1 (8)	11	6671	35 893
*HGSNAT*	1 (2)	61	5214	62 392
*NEUROD1*	1 (3)	36	3002	12 533
*ZNF513*	1 (100)	0	2158	3556
*AHR*	1 (20)	4	6243	429 794
*ARHGEF18*	1 (33)	2	5741	131 053
*MVK*	1 (0)	164	2073	24 871
*NEK2*	1 (20)	4	2134	17 375
*SLC17A4*	1 (33)	2	3716	26 501
Total	2092	3776	255 701	6 944 411

The mutations data were extracted from the HGMD Pro database. Data were presented as numbers (N), percentages (%) and frequencies. The percentages are the proportion of all mutations at each locus that are arRCD mutations.

The total exons size was calculated by adding the length of every exon in a gene (LoVD database: https://grenada.lumc.nl/LSDB_list/lsdbs/). Gene size were retrieved from GeneCards (www.genecards.org)

arRCD, autosomal recessive rod-cone dystrophy; HGMD, Human Gene Mutation Database.

The mutations occurrence in arRCD and other diseases is provided in table 1. Only 34% of the total mutations in *USH2A* were known to cause arRCD. In contrast, >90% of *RP1, EYS*, *CNGB1* and *PDE6A* mutations were causing arRCD (table 1). Mutations in *RLBP1*, *RP1L1*, *CRB1* and *PROM1* were moderately implicated (41%–43%, table 1). Although*ABCA4* had the highest number of mutations, only 5% of its mutations were arRCD. Other genes such as *BEST1*, *HGSNAT* and *NEUROD1* have smaller implications (<5%, table 1). We tested the possibility that longer genes have more arRCD mutations. One would anticipate that arRCD is a deleterious trait and that the accumulation of mutations should be proportional to the number of (functional) base pairs. The correlation analysis between gene size and the pattern of arRCD mutations showed no associations (p>0.05).

To investigate possible mutational signatures for arRCD, we have stratified the different types of mutations according to arRCD. We found that the mutations’ types varied according to the phenotype (non-arRCD and arRCD) (table 2, p≤0.004). Specifically, we observed a 5% decrease in missense (61% in non-arRCD vs 56% in arRCD), an increase in InDels (23% in non-arRCD vs 25% in arRCD) and nonsense mutations (15% in non-arRCD vs 16% in arRCD) (table 2). These results were replicated in the LOVD database since the variation types showed a similar trend (table 2). The quantification of these associations showed that InDels (O.R=1.2 and p=0.007, table 2), nonsense (O.R=1.2 p=0.014, table 2) and splice site (O.R=1.6 and p=0.038, table 2) increased the OR of developing arRCD by 20%–60% compared with missense in HGMD. Similar trends were also seen in the LOVD database (table 2). Importantly, when the associations between the mutations’ type and USHER syndrome were conducted, we found that the InDels, nonsense and splice-site mutations increased the OR of USHER syndrome at least twice when compared with missense (2<OR<2.4, p<0.0001, table 2).

**Table 2:** Association of the total mutations with autosomal recessive rod-cone dystrophy

	HGMD pro								LOVD					
	arRCD versus non-arRCD						arRCD versus USHER syndrome		arRCD versus non-arRCD					
Mutation	Non-arRCD	arRCD	X^2^	P value*	OR (95% CI)	P value†	OR (95% CI)	P value‡	Non-arRCD	arRCD	X^2^	P value*	OR (95% CI)	P value†
Missense	61%	56%	15	0.004	1		1		51%	34%	17	0.001	1	
Indel	23%	25%			1.2 (1.1 to 1.4)	0.007	2.10 (1.73 to 2.55)	<0.0001	29%	36%			1.9 (1.1 to 3.3)	0.017
Nonsense	15%	16%			1.2 (1 to 1.4)	0.014	2.39 (1.92 to 2.96)	<0.0001	17%	20%			1.7 (0.9 to 3.2)	0.09
Splice Site	1%	2%			1.6 (1 to 2.4)	0.038	2.15 (1.64 to 2.8)	<0.0001	3%	9%			5.3 (2.2 to 13)	0.0001

OR: measure of association between an exposure and an outcome.

χ^2^ measure of association between two categorical variable variables (related or independent).Regulatory mutations were not shown in the table because of the low sample size.

*P value for X^2^ test was used to compare the mutations' occurrence in non-arRCD versus arRCD.

†P value for multiple logistic regression analysis of genetic mutations with arRCD (arRCD vs non-arRCD).

‡P value for multiple logistic regression analysis of genetic mutations with arRCD (arRCD vs Usher syndrome).

arRCD, autosomal recessive rod-cone dystrophy; HGMD, Human Gene Mutation Database.

We have conducted a gene-based analysis and found enrichment for arRCDamong eight genes: *ABCA4, BBS1, CRB1, CYP4V2, EYS, IMPG2, RP1L1* and *USH2A* (χ^2^, p<0.05, table 3). Mutations in these genes were distributed differently between arRCD and non-arRCD. In *ABCA4, BBS1, CYP4V2*, >92% of missense, InDels and nonsense mutations were enriched in non-arRCD disorders (p≤0.043, table 3). In *CRB1*, nonsense and InDels mutations were enriched in non-arRCD (p=0.0001, table 3). In *EYS*, all types of mutations were enriched in arRCD (p=0.002). In *IMPG2*, all the InDels and splice sites were enriched in arRCD, whereas two-thirds of the missense mutations were non-arRCD (p=0.002, table 3). In *RP1L1*, the majority of the nonsense mutations were arRCD (p=0.05, table 3). In *USH2A*, the InDels, nonsense and splice site mutations belonged mainly to the group ‘non-arRCD’ (p=0.0001, table 3), while the missense mutations did not show any preference (~50%).

**Table 3:** Gene mutations pattern according to autosomal recessive rod-cone dystrophy

Gene	Mutation	Non-arRCD	arRCD	X^2^	P value
*ABCA4*	Missense	774 (95%)	41 (5%)	8	0.043
	InDel	219 (94%)	13 (6%)		
	Nonsense	145 (95%)	7 (5%)		
	Splice Site	1 (50%)	1 (50%)		
	Total (N)	1109	62		
*BBS1*	Missense	39 (93%)	3 (7%)	9	0.031
	InDel	27 (96%)	1 (4%)		
	Nonsense	17 (100%)	0		
	Splice Site	1 (50%)	1 (50%)		
	Total (N)	84	5		
*CRB1*	Missense	98 (45%)	120 (55%)	34	0.0001
	InDel	50 (75%)	17 (25%)		
	Nonsense	46 (81%)	11 (19%)		
	Splice Site	1 (50%)	1 (50%)		
	Total (N)	195	149		
*CYP4V2*	Missense	63 (94%)	4 (6%)	8	0.021
	Nonsense	14 (100%)	0		
	Splice Site	1 (50%)	1 (50%)		
	Total (N)	78	5		
*EYS*	Missense	8 (5%)	146 (95%)	15	0.002
	InDel	4 (3%)	111 (97%)		
	Nonsense	0	67 (100%)		
	Splice Site	1 (50%)	1 50%)		
	Total (N)	13	325		
*IMPG2*	Missense	9 (64%)	5 (36%)	15	0.002
	InDel	0	11 (100%)		
	Nonsense	3 (19%)	13 (81%)		
	Splice Site	0	2 (100%)		
	Total (N)	12	31		
*RP1L1*	Missense	29 (66%)	15 (34%)	6	0.05
	InDel	3 (60%)	2 (40%)		
	Nonsense	4 (22%)	7 (78%)		
	Total (N)	34	24		
*USH2A*	Missense	355 (54%)	306 (46%)	100	<0.001
	InDel	261 (81%)	60 (19%)		
	Nonsense	194 (80%)	48 (20%)		
	Splice Site	1 (75%)	1 (25%)		
	Total (N)	811	415		

The mutations data were extracted from the HGMD Pro database. Data were presented as numbers (N) and percentages (%).

P value for χ^2^ test was used to compare the mutations’ occurrence in non-arRCD versus arRCD.

χ^2^ measure of association between two categorical variable variables (related or independent).

arRCD, autosomal recessive rod-cone dystrophy; HGMD, Human Gene Mutation Database; InDel, insertion/deletion.

To go further, we searched for specific coding exons harbouring arRCD mutations (table 4). Our analysis revealed that exons 20 and 28 coding for the cytoplasmic region between nucleotide binding domains (NBD) and transmembrane domain (TMD, 1007 a.a-1,051 a.a) and the extracellular domain (ECD, 1411 a.a-1,452 a.a) in *ABCA4* belonged to the group ‘non-arRCD’. In contrast, all the coding exons in *EYS* were enriched in arRCD (table 4). Exons 2, 6 and 7 in *CRB1* coding for EGF Like and Laminin G Like domains contain InDels and nonsense mutations that are non-arRCD (p<0.05, table 4). Interestingly, mutations in exon 4 of *RP1L1* showed an opposite pattern: the InDels were non-arRCD’, whereas the nonsense mutations showed an enrichment in arRCD (p=0.001, table 4). Mutations in exons 12, 60 and 62 coding for Laminin EGF and FTIII domains of *USH2A* showed a different spectrum: the missense mutations were mainly arRCD, whereas the InDels, nonsense and splice site were enriched in non-arRCD (p=0.001, table 4).

**Table 4:** Association of exons and protein domains with autosomal recessive rod-cone dystrophy

Gene	mRNA refseq	Exon	Amino acids	Domain	Type	Phenotype		X^2^	P value
						Non-arRCD	arRCD		
*ABCA4*	NM_000350.3	20	1007–1051	Cytoplasmic region between NBD and TMD	Missense	16 (100%)	0	8	0.047
					InDel	5 (100%)	0		
					Nonsense	2 (67%)	1 (33%)		
					Splice site	2 (100%)	0		
					Total (N)	25	1		
		28	1411–1452	ECD	Missense	19 (100%)	0	10	0.021
					InDel	2 (50%)	2 (50%)		
					Nonsense	3 (100%)	0		
					Splice site	10 (84%)	2 (16%)		
					Total (N)	34	4		
*EYS*	NM_001142800.2	1–44	All	Signal peptide, EGF, EGF Ca Binding, Laminin G	Missense	8 (5%)	146 (95%)	58	0.011
					InDel	4 (4%)	109 (96%)	58	0.0001
					Splice site	1 (4%)	27 (96%)	28	0.032
					Total (N)	13	325		
*CRB1*	NM_201253.3	2	77–271	EGF-like 2–3 to EGF-like 4–7; calcium-binding	Missense	7 (39%)	11 (61%)	10	0.017
					InDel	11 (85%)	2 (15%)		
					Nonsense	5 (100%)	0		
					Splice site	1 (50%)	1 (50%)		
					Total (N)	24	14		
		6	445–763	EGF-like 11–12 and Laminin G-like 1–2	Missense	29 (49%)	30 (51%)	7	0.05
					InDel	10 (71%)	4 (29%)		
					Nonsense	14 (82%)	3 (18%)		
					Splice site	2 (40%)	3 (60%)		
					Total (N)	55	40		
		7	764–946	EGF-like 13–14 and Laminin G-like 2	Missense	19 (44%)	24 (56%)	14	0.004
					InDel	15 (88%)	2 (12%)		
					Nonsense	8 (89%)	1 (11%)		
					Splice Site	1 (50%)	1 (50%)		
					Total (N)	43	28		
*RP1L1*	NM_178857.6	4	339–2671	Repeated domains	Missense	22 (73%)	8 (27%)	9	0.001
					InDel	2 (100%)	0%		
					Nonsense	2 (22%)	7 (78%)		
					Total (N)	26	15		
*RPE65*	NM_000329.3	9	303–349	–	Missense	10 (91%)	1 (9%)	9	0.032
					InDel	3 (100%)	0		
					Nonsense	3 (100%)	0		
					Splice site	0	1 (100%)		
					Total (N)	16	2		
*USH2A*	NM_206933.4	12	869–1083	Laminin EGF	Missense	7 (37%)	12 (63%)	18	0.0001
					InDel	12 (100%)	0		
					Nonsense	7 (88%)	1(13%)		
					Splice site	4 (100%)	0		
					Total (N)	30	13		
		60	4050–4168	FTIII	Missense	7 (37%)	12 (63%)	11	0.001
					InDel	13 (93%)	1 (7%)		
					Nonsense	2 (67%)	1 (33%)		
					Splice site	3 (75%)	1 (25%)		
					Total (N)	25	15		
		62	4244–4750	FTIII	Missense	30 (42%)	42 (58%)	16	0.001
					InDel	26 (74%)	9 (26%)		
					Nonsense	19 (79%)	5 (21%)		
					Splice site	2 (67%)	1 (33%)		
					Total (N)	77	57		

The mutations data were extracted from the HGMD Pro database. Data were presented as numbers (N) and percentages (%).

P value for χ^2^ test was used to compare the mutations’ occurrence in non-arRCD versus arRCD.

χ^2^ measure of association between two categorical variable variables (related or independent).

arRCD, autosomal recessive rod-cone dystrophy; ECD, extracellular domain; InDel, insertion/deletion; NBD, nucleotide binding domain; TMD, transmembrane domain.

## Discussion

Here, we found that 36% of all the downloaded mutations in arRCD genes were specific for arRCD. The remaining two-thirds were present in non-arRCD phenotypes such as Stargardt disease, Usher syndrome and Leber congenital amaurosis and non-retinal phenotypes such as hearing impairment and diabetes. We showed that the mutations’ pattern differed according to arRCD than non-arRCD. Compared with missense, InDels, nonsense and splice-site mutations increased the ORs of arRCD by 20%–60% versus non-arRCD. Furthermore, we have conducted a gene-based analysis and found enrichment for *EYS, IMPG2, RP1L1* and *USH2A mutations* with arRCD. The exon-based analysis revealed that the vast majority of *EYS* mutations were enriched for arRCD. In contrast, the mutations in *RP1L1* exon 4 and *USH2A* exons 12, 60 and 62 showed opposite patterns; the missense mutations were mainly arRCD, whereas the InDels, nonsense and splice site were specific for non-arRCD.

The investigation of the mutational spectrum in arRCD genes differed between arRCD and non-arRCD conditions. Specifically, the prevalence of nonsense, InDels and splice-site mutations increased in arRCD. Furthermore, these types had a higher OR of arRCD (20%–60%). Noteworthy, these results were replicated in the LOVD database since the latter showed the same trend observed in the HGMD database.

Our findings point out that the InDels, nonsense and splice-site mutations increased the OR of USHER syndrome at least twice compared with missense (table 2). These findings go in the same direction with: (1) a survey of targeted panel sequencing in 525 Japanese RCD patients revealed that truncating variants in *USH2A* were detected in all syndromic patients with more severe phenotypes than non-syndromic ones;^11^ (2) in the largest cohort of Chinese patients with *USH2A*, Zhu *et al* reported that individuals with truncating mutations experienced an earlier decline in visual function.^12^ All the above is reasonable since truncating mutations might largely inactivate the function of the entire protein, thus leading to a more severe phenotype.

The differences in the mutation types between arRCD and non-arRCD phenotypes, directed us to a gene-based analysis to identify the genes responsible for these differences. Of note, this analysis revealed that unlike *EYS, CNGB1* and *PDE6A* whose functions appear to be uniquely tied to arRCD, genes such as *ABCA4* and *USH2A* seem to play a broader physiological role because mutations in it are commonly associated with conditions other than just arRCD. A possible explanation for being arRCD ‘specific’ is their expression site, as they are predominantly expressed in the eye and specifically involved in the biology of rods and cones. RP1 is a microtubule-associated protein crucial for photoreceptors' function, It encodes a photoreceptor-specific protein expressed in rods and cones.^2 13^ *EYS* is the largest gene expressed in the human eye. It is expressed in the human retina with minor expression in other tissues.^14^ Human EYS has been shown to play a role in stabilising ciliary axonemes in rods and cones photoreceptors.^15^ In humans, *CNGB1* encodes an ion channel needed for phototransduction by regulating the ion flow into the rod photoreceptor in response to light-induced alterations.^16^ Mutations in *PDE6A* lead to excessive accumulation of cGMP and subsequent rod, followed by cone photoreceptors death.^17^

*USH2A* has been shown to harbour mutations causing Usher syndrome type II and non-syndromic arRCD. Mutations in exons 12, 60 and 62 coding for Laminin EGF and FTIII domains in USH2A showed an interesting pattern of implication in arRCD: missense mutations were mainly arRCD specific, whereas InDels, nonsense and splice site mutations were abundant in non-arRCD. The mechanisms involved in this phenotypic variation are usually presented as assumptions and only exceptionally rely on proven data.^18^ The phenotype heterogeneity associated with *USH2A* mutations underlines the complex relationship between the disease-causing mutations and the retinal phenotype, rendering the Mendelian concept of monogenic diseases not applicable for a growing number of diseases.

For decades, the genotype–phenotype correlations were based on cosegregation analysis inside small pedigrees. Our study is the first to use statistical tests to investigate the mutations patterns globally, per gene and per exons according to arRCD. On the other hand, this study has several limitations: (1) Our analysis relied on the number of reported mutations and not the patients carrying them; thus, we could not use the allelic frequencies in all our analysis; (2) No association with specific clinical ocular phenotypes such as the visual field, the electroretinogram and the fundus appearance was performed; (3) We could not stratify these genotype–phenotype correlations according to the geographical location and (4) For the gene based, one test per gene was performed (63 independent tests in total); thus, a Bonferroni correction might further be used. If applied, USH2A and CRB1 remain highly associated (p<0.001). In the exon-based analysis, one test was performed for all the gene exons, thus abolishing the concern of multiple testing.

In conclusion, the current approach showed specific mutational patterns specifically enriched in arRCD.

## Data Availability

Data are available in a public, open access repository. The raw data of the current study was deposited in DRYAD repository: https://datadryad.org/stash/share/9ntbTjE-nvOl_mB1P-9UamV0k2NcAennp6NvIm5yqDw. No licence is needed.
